# Solid-state esophageal pressure sensor for the estimation of pleural pressure: a bench and first-in-human validation study

**DOI:** 10.1186/s13054-025-05279-w

**Published:** 2025-01-27

**Authors:** Julien P. van Oosten, Nico Goedendorp, Amne Mousa, Rutger C. Flink, Rik Schaart, Merel Flinsenberg, Peter Somhorst, Diederik A. M. P. J. Gommers, Leo Heunks, Annemijn H. Jonkman

**Affiliations:** 1 Intensive Care, Erasmus Medical Center, Dr. Molewaterplein 40, 3015 GD Rotterdam, The Netherlands; 2 Intensive Care, Amsterdam UMC Location Vrije Universiteit Amsterdam, Amsterdam, The Netherlands; 3 Amsterdam Cardiovascular Sciences, Amsterdam, The Netherlands; 4 Pulmotech B.V, Leek, The Netherlands; 5 Intensive Care, Radboud University Medical Center, Nijmegen, The Netherlands

**Keywords:** Solid-state sensor, Esophageal catheter, Respiratory monitoring, Pleural pressure, Mechanical ventilation

## Abstract

**Background:**

Advanced respiratory monitoring through the measurement of esophageal pressure (Pes) as a surrogate of pleural pressure helps guiding mechanical ventilation in ICU patients. Pes measurement with an esophageal balloon catheter, the current clinical reference standard, needs complex calibrations and a multitude of factors influence its reliability. Solid-state pressure sensors might be able to overcome these limitations.

**Objectives:**

To evaluate the accuracy of a new solid-state Pes transducer (Pes_solid_). We hypothesized that measurements are non-inferior to those obtained with a properly calibrated balloon catheter (Pes_bal_).

**Methods:**

Absolute and relative solid-state sensor Pes measurements were compared to a reference pressure in a 5-day bench setup, and to simultaneously placed balloon catheters in 15 spontaneously breathing healthy volunteers and in 16 mechanically ventilated ICU patients. Bland–Altman analysis was performed using mixed effects modelling with bootstrapping to estimate bias and upper and lower limits of agreement (LoA) and their confidence intervals.

**Results:**

*Bench study*: Solid-state pressure transducers had a positive bias (P_solid_ – P_ref_) of around 1 cmH_2_O for the absolute minimal and maximum pressures, and no bias for pressure swings. *Healthy volunteers:* the solid-state transducer revealed a bias (i.e._,_ Pes_solid_ – Pes_bal_) [upper LoA; lower LoA] of 1.59 [8.21; − 5.02], − 2.32 [4.27; − 8.92] and 3.91 [11.04; − 3.23] cmH_2_O for end-expiratory, end-inspiratory and ΔPes values, respectively. *ICU patients:* the solid-state transducer showed a bias (Pes_solid_–Pes_bal_) [upper LoA; lower LoA] during controlled/assisted ventilation of: − 0.15 [1.41; − 1.72]/− 0.19 [5.23; − 5.62], 0.32 [3.45; − 2.82]/− 0.54 [4.81; − 5.90] and 0.47 [3.90; − 2.96]/0.35 [4.01; − 3.31] cmH_2_O for end-expiratory, end-inspiratory and ΔPes values, respectively. LoA were ≤ 2cmH_2_O for static measurements on controlled ventilation.

**Conclusions:**

The novel solid-state pressure transducer showed good accuracy on the bench, in healthy volunteers and in ventilated ICU-patients. This could contribute to the implementation of Pes as advanced respiratory monitoring technique.

**Trial registration:**

Clinicaltrials.gov identifier: NCT05817968 (patient study). Registered on 18 April 2023.

**Supplementary Information:**

The online version contains supplementary material available at 10.1186/s13054-025-05279-w.

## Introduction

Advanced respiratory monitoring through the measurement of esophageal pressure (Pes) as a surrogate for pleural pressure helps understanding partitioned respiratory mechanics and breathing effort in mechanically ventilated patients, and could guide the individualization of ventilator settings [1]. Despite the presumed benefits for the individual patient’s management, implementation of esophageal manometry is still in its infancy [1–5]. It requires (technical) expertise and the validation and calibration of balloon catheters is often challenging. For instance, optimal filling volumes vary according to the balloon type, patient factors and ventilator settings, and both excessive and insufficient balloon filling volumes dampen Pes amplitudes [1, 6]. In addition, the balloon may empty over time, resulting in an underestimation of pressures. Signal dampening could also occur if compliant tubing is used to connect the catheter to the extracorporeal pressure sensor. Therefore, for correct interpretation of respiratory physiology and optimal patient/ventilator management, adequate balloon position and filling volume should be regularly confirmed with an occlusion test and adjusted accordingly [1].

Pes catheters using a solid-state pressure transducer might be able to overcome some of the above limitations. These sensors measure Pes directly inside the esophagus, allowing a faster frequency response while not being subjected to signal dampening. Previous older studies have used such transducers, but showed unacceptable signal drifting [7, 8]. These studies, however, used pressure transducers that were not correctly (temperature) calibrated. Here, we evaluate the accuracy of a new solid-state Pes catheter with a transducer that allows for both temperature and ambient pressure calibration. We hypothesized that measurements are non-inferior as compared to a correctly calibrated balloon catheter. We tested this hypothesis in a bench setup, in healthy volunteers, and in mechanically ventilated patients during controlled and assisted ventilation.

## Methods

For additional details, see Supplementary material 1.

### Study design and subjects

This study consisted of: 1) a bench study (September 2023) at the manufacturer location (Pulmotech B.V., Leek, the Netherlands), 2) a prospective study in spontaneously breathing healthy volunteers (August–October 2021) at the intensive care unit (ICU) of the Amsterdam University Medical Center, Amsterdam, the Netherlands (ethics approval number METC 2020.470), and 3) a prospective study in mechanically ventilated ICU patients (October 2023 to March 2024) at the ICU of the Erasmus Medical Center, Rotterdam, The Netherlands (ethics approval number MEC-2023–0119; ClinicalTrials.gov: NCT05817968). Extensive technical development tests were performed prior to the healthy volunteer study; these data are not part of this manuscript. Written informed consent was obtained from healthy subjects and patients according to local regulations.

### Healthy volunteers

We recruited healthy, non-obese adults without history of cardiac and/or pulmonary disease and contraindications for nasogastric catheter placement (e.g., esophageal varices, recent (< 2 weeks) nasal bleeding, use of anticoagulants).

### Patients

Elective adult cardiothoracic surgery patients requiring postoperative invasive controlled mechanical ventilation in the ICU were enrolled pre-surgery. Eligibility was reassessed at ICU arrival. Exclusion criteria were: (1) upper airway/esophageal/mouth or face pathology (i.e. recent surgery, esophageal varices, diaphragmatic hernia), (2) nasal bleeding within the last 2 weeks, (3) presence of pneumothorax, (4) inadequate coagulation, (5) pregnancy.

### Data collection

We collected sex, age, height, body mass index (BMI), and for the ICU population also the relevant medical history (cardiac and pulmonary diseases), type of surgery performed, vital signs and ventilator settings throughout study procedures. Device-related adverse events were noted.

### Esophageal manometry

We tested the intelligent Esophageal Pressure Catheter (iEPC) (PulmoTech B.V., Leek, The Netherlands), a CE-marked 12 French catheter with a length of 125cm that combines nasogastric feeding with Pes measurements via a solid-state pressure sensor (Supplementary material 2). The catheter was connected to an acquisition system (iEPMS, PulmoTech B.V., Leek, The Netherlands, connected to Polybench, Applied Biosignals GmbH, Weener, Germany) for data sampling at 200Hz.

Measurements were compared with a standard balloon catheter, either the Cooper catheter (Cooper Surgical, Trumbull, USA: 5 French, length 85cm, balloon length 9.5cm – used in healthy volunteers and patients) or the NutriVent™ catheter (Sidam group, Mirandola, Italy: 14 French catheter, length 108cm, balloon length 10cm – used in patients). The balloon catheter, an airway pressure (Paw, healthy subjects + patients) and flow sensor (healthy subjects) were connected to an acquisition system (MP160, BIOPAC Systems Inc., Goleta, USA) for simultaneous recording of waveforms sampled at 200Hz. Waveforms were synchronized with the solid-state sensor tracings offline.

### Procedures

#### Bench

Catheters were exposed to physiological conditions (100% relative humidity and 37°C) for 5 days (Supplementary material 3). Pressure swings of 12 cmH_2_O above 10 cmH_2_O baseline at a rate of 12/minute were applied using the AVEA (Viasys Healthcare, Conshohocken, USA) in conjunction with a humidifier (Fisher&Paykel MR850, Auckland, New Zealand). Temperature and relative humidity were controlled to maintain 37 ± 2°C and > 90%, respectively. Reference pressure (P_ref_) was measured through a non-compliant tube connected to the setup, calibrated and validated against an accredited test device (PACE5000, General Electric, Fairfield, USA). Multiple catheters were measured at once (two runs of 8 catheters each) for 5 days, impacting the maximum possible sample rate of the acquisition system/software. Therefore, data was recorded at 30Hz for the solid-state pressures (P_solid_), and at 200Hz for P_ref_ (iEPMS, PulmoTech B.V.). Since the objective of the bench test was to evaluate drift of the sensor over five days, the lower sample rate was not an issue.

### Healthy volunteers

#### Catheter placement/calibration

Prior to insertion, the solid-state sensor was calibrated according to the manufacturer’s instructions (see Supplementary material 1). Both catheters were inserted aimed at measuring Pes in the mid-esophageal range; location of the sensor corresponded to approximately halfway the balloon. Esophageal placement was confirmed by cardiac artifacts/esophageal spasms on the pressure waveforms. Balloon filling volume was per manufacturer’s instructions (Cooper: inflate with 2.0 ml, then remove 1.5 ml) checked with the Baydur maneuver (end-expiratory occlusion test) and adjusted when needed (see Supplementary material 1). The ΔPes_bal_/ΔPaw ratio was targeted at 0.9–1.1 for higher accuracy. If this range was not reached after five maneuvers, a range of 0.8–1.2 was accepted.

#### Measurements

Recordings were obtained with subjects in sitting, semi-recumbent, supine and prone position. Balloon catheter accuracy was verified in between position changes. In each body position, 2 min of Pes during unloaded tidal breathing was acquired. During sitting and semi-recumbent position, subjects were additionally shortly exposed to three levels of inspiratory effort to obtain a variable within-subject range of effort (thus Pes values), using a threshold loading device (Power Breathe, POWERbreathe Ltd, Warwickshire, UK), see Supplementary material 1.

### Patients

The study protocol was initiated directly after surgery upon arrival on the ICU. Ventilator settings were according to clinical protocols.

#### Catheter placement/calibration

Catheter positioning and calibration were similar to healthy volunteers, while patients were still deeply sedated. Position was verified with thoracic X-ray when made within standard of care and/or using video laryngoscopy. The NutriVent catheter was initially used as comparator; however, in the first four patients, interference between both catheters was observed (see Results). From the 5th patient, the Cooper catheter was used instead.

#### Measurements

During controlled ventilation, 10 min of tidal breathing were recorded, and three end-inspiratory and end-expiratory holds were performed (at 0, 5 and 10 min) for static measurements. Another 10-min recording was performed during partially assisted ventilation when spontaneous breathing resumed as per clinical care. Correct balloon filling volume was verified at the start of each recording. Catheters were removed upon study completion.

### Offline analysis

For the bench study, the minimum, maximum and delta pressures were calculated using custom software (Polybench, Applied BioSignals, Weener, Germany). At each measurement time point, a median for each parameter was calculated over 60 preceding artificial breaths for further analyses.

For the healthy volunteers and patient studies, signal processing and analyses were performed in MATLAB (Mathworks, Natick, USA). Periods or individual breaths with substantial artifacts (e.g., esophageal spasms, coughing) were removed. Signals were processed using a 2nd-order 5 Hz low-pass Butterworth filter followed by a 0.1 s unweighted moving average filter. Static measurements for end-expiratory and end-inspiratory holds (in patients) and the Baydur maneuver (ΔPes_bal_/ΔPaw and ΔPes_solid_/ΔPaw) were manually selected from the tracings. Whereas the ΔPes_bal_/ΔPaw was used to verify balloon filling volume throughout the study, for the solid-state pressure transducer the Baydur test served as offline measure for sensor stability (as this catheter only requires zeroing before insertion). A breath detection algorithm was used and absolute values for end-inspiratory Pes, end-expiratory Pes and the resulting inspiratory amplitude (ΔPes) were computed breath-by-breath for both signals (in cmH_2_O).

### Endpoints

For the bench study, P_solid_ was compared to P_ref_. For healthy subjects and patients, the primary endpoint was the difference in absolute Pes values between the solid-state sensor and balloon catheter (Pes_solid_–Pes_bal_), measured at end-expiration and peak inspiration, and the difference in relative Pes values (i.e., inspiratory amplitude) between both catheters (∆Pes_solid_–∆Pes_bal_). Endpoints were separated for the different ventilation modes/populations. Secondary endpoints were the stability of the solid-state catheter as from repeated Baydur values, and device-related adverse events.

### Sample size

Sixteen catheters were tested on the bench; a convenience sample based on standard deviations (SD) obtained in the manufacturer’s previous technical tests. These tests were also used to substantiate sample sizes for the in-human studies, resulting in 7 subjects required for Bland–Altman analyses for each study, assuming a type-I error of 0.05 and type-II error of 0.20, and the following variables: expected mean (SD) of difference: 0.79 (0.53) cmH_2_O; maximum allowed difference between methods: 3.5 cmH_2_O, based on pressure accuracy tests and sensor drift tests (Pulmotech B.V., Leek, The Netherlands). We enrolled a larger sample (15 healthy subjects, 16 patients) to allow for more variability, to increase user-experience and to account for potential clinical/technical challenges.

### Statistical analysis

Statistical analyses were performed in R (version 4.4.1, R Foundation for Statistical Computing, Vienna, Austria). For the healthy volunteer and patient studies, baseline demographics and/or ventilator settings are presented as median (interquartile range, IQR) or numbers (%). Different analyses were performed (see Supplementary material 1 for details):

1. For the bench tests (11 time points), bias (i.e., Pes_solid_ – Pes_ref_), SD of the differences and upper and lower limits of agreement (LoA, i.e., bias ± 1.96 SD) were computed per measurement time point, separately for the minimum, maximal and delta pressures. Linear mixed effects models were used to obtain within-sensor variability (i.e., residual SD) and between-sensor variability (i.e., random effects SD) of the bias over the full study period.
2. For the breath-by-breath analysis in healthy subjects and patients, all breaths per subject were included and analyses were done for the end-expiratory Pes, end-inspiratory Pes and ∆Pes separately. Bland–Altman analysis was performed via linear mixed effects models to correct for the repeated measures design and with bootstrapping (1000 iterations) to account for the variable number of breaths across subjects/patients (e.g., due to varying respiratory rate and included breaths with sufficient signal quality). We computed the bias (Pes_solid_ – Pes_bal_), SD of the differences and 95% lower and upper LoAs, as well as the 95% confidence interval of these metrics.
3. For the static measurements in patients (three end-expiratory and end-inspiratory holds during controlled ventilation), the bias (i.e., Pes_solid_ – Pes_bal_), SD of differences and upper and lower LoAs were calculated with linear mixed effects models to take into account the repeated measures design, but without bootstrapping as the number of data points was identical between patients.
4. For the healthy volunteers where variability in inspiratory effort was introduced with loaded breathing, agreement between breath-by-breath Pes values for the solid-state vs. balloon catheter was also evaluated with a linear mixed effects model to obtain a conditional R^2^ accounting for differences in the number of repeated measures between subjects. Individual subject correlations were evaluated with simple linear regression.

## Results

### Bench study

**Fig. 1:**
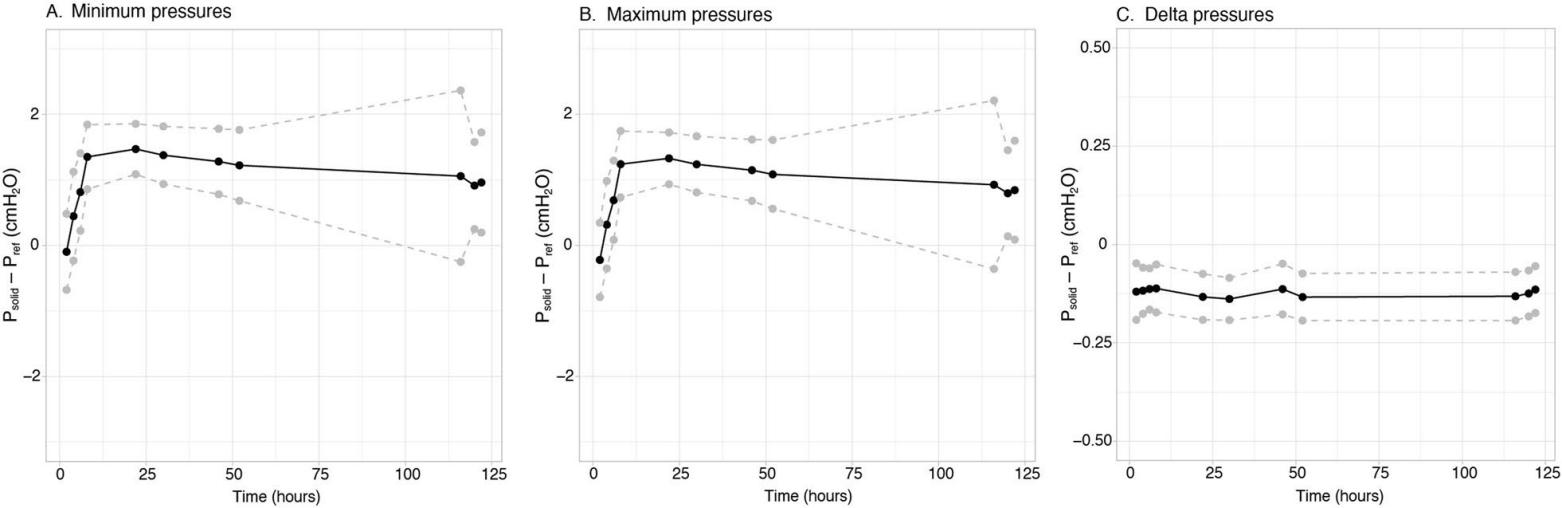
Bench results over a 120-h measurement period, for the minimum pressures (**A**), maximum pressures (**B**) and delta pressures (**C**). Black line represents the mean difference (bias, i.e. solid-state catheter pressure (Psolid) minus reference pressure (Pref)), dashed gray lines are the upper and lower limits of agreement

The 16 solid-state sensors demonstrated a small positive bias (P_solid_–P_ref_) that increased from 0 to approximately 1.5 cmH_2_O during the first 10 h for the absolute minimal and maximum pressures, and then remained stable at around 1 cmH_2_O until 120 h (Fig. 1A,B). There was a negligible bias for pressure swings (0.13 cmH_2_O) throughout the full study (Fig. 1C). Within-sensor and between-sensor variability of the bias over the full study period were 0.53 and 0.16 cmH_2_O for minimal pressures, 0.53 and 0.16 cmH_2_O for maximum pressures, and 0.02 and 0.03 cmH_2_O for pressure swings, respectively.

### Healthy volunteers

Fifteen healthy subjects (male/female 3/12; age 35.5 ± 13.5 years) completed the study without adverse events. Two subjects were excluded from the full analysis, because of balloon catheter dislocation early in the study and inability to obtain reliable recordings after this was noticed (n = 1), or balloon Baydur (∆Pes_bal_/∆Paw) exceeding the 0.8–1.2 range throughout the study (n = 1). For other subjects (n = 5), short sections with low signal quality were removed (i.e., many esophageal spasms and/or cardiac artefacts, or ∆Pes_bal_/∆Paw not within 0.8–1.2 range for a specific body position).

### Comparisons

**Fig. 2:**
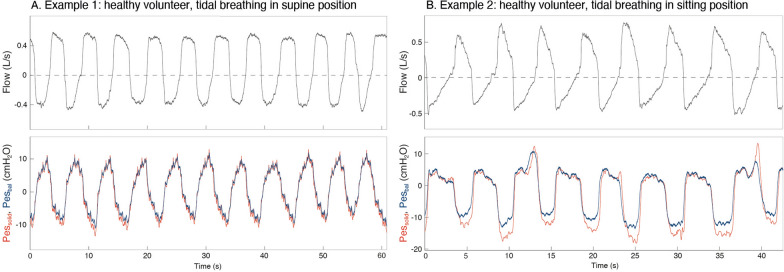
Examples of tracings obtained in two different healthy volunteers, during unloaded tidal breathing in supine (**A**) and sitting (**B**) position

**Fig. 3:**
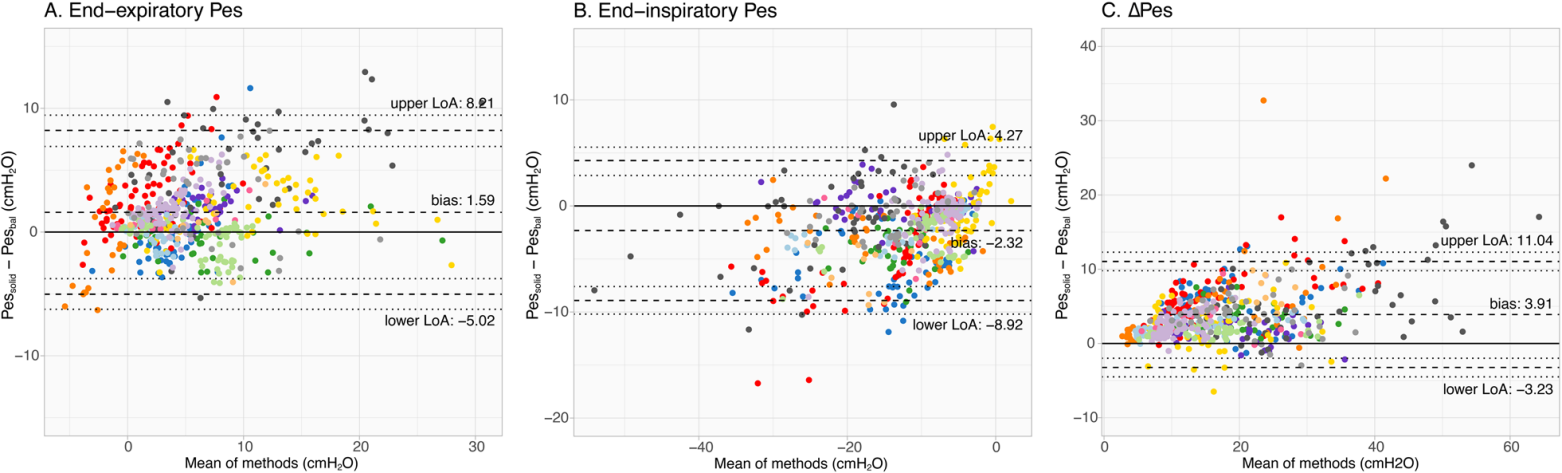
Bland–Altman results for healthy volunteers for the end-expiratory (**A**), end-inspiratory (**B**) and delta (**C**) Pes values. All individual breaths were gathered for each subjects, independent of the loading condition and/or body position (that was done to generate a physiological range of Pes values). Each color represents a different subject. Dashed lines represent the bias and upper and lower limits of agreement (LoA); dotted lines represent the 95% confidence interval of the LoA obtained via bootstrapping

Figure 2 shows examples of Pes_bal_ and Pes_solid_ tracings. A total of 563 breaths of thirteen subjects were included in analyses when accepting tracings with ΔPes_bal_/ΔPaw within the 0.9–1.1 range. Bland–Altman analyses (Fig. 3, Supplementary material 1: Table S1-A) revealed a bias (i.e._,_ Pes_solid_ – Pes_bal_) [upper LoA; lower LoA] of 1.59 [8.21; − 5.02], − 2.32 [4.27; − 8.92] and 3.91 [11.04; − 3.23] cmH_2_O for end-expiratory, end-inspiratory and ΔPes values, respectively. Pes_bal_ and Pes_solid_ values showed good to excellent correlations (Supplementary materials 4, 5, 6), with a conditional R^2^ obtained with linear mixed effects modeling of 0.89, 0.92 and 0.93 for end-expiratory, end-inspiratory and ΔPes values, respectively.

The average ΔPes_bal_/ΔPaw was 0.87 ± 0.26 (min–max: 0.39–1.12; n = 97 maneuvers), indicating that the balloon required frequent recalibrations after e.g. body position changes before adequate comparisons with Pes_solid_ could be made. For the solid-state catheter, Baydur maneuvers were analyzed offline to determine sensor stability. Of the 98 maneuvers analysed, ΔPes_solid_/ΔPaw ratio was 1.01 ± 0.14 [min–max: 0.74–1.54]; 10 measurements (of which 4 from one subject) exceeded the 0.8–1.2 range.

We performed two sensitivity analysis: 1) when accepting tracings with ΔPes_bal_/ΔPaw within the 0.8–1.2 range, 877 breaths were included, resulting in comparable bias and LoA (Supplementary material 1: Table S1-B, Supplementary material 7); 2) when selecting only tracings with both ΔPes_solid_/ΔPaw and ΔPes_bal_/ΔPaw ratios between 0.9–1.1 and excluding one subject where cardiac artifacts amplitudes exceeded 5 cmH_2_O in both signals, 357 breaths from 11 subjects were used, resulting in a lower bias [upper LoA; lower LoA]: 0.70 [6.95; − 5.55], − 2.12 [3.38; − 7.62] and 2.86 [8.52; − 2.80] cmH_2_O for end-expiratory, end-inspiratory and ΔPes values, respectively (Supplementary material 1: Table S1-C).

### Patients

27 patients consented prior to their surgery, and 16 patients (see Table 1 for characteristics) were eventually enrolled upon ICU arrival. Reasons for withdrawal were a last-minute canceled/rescheduled surgery (n = 10) and hemodynamic instability (n = 1). One patient was excluded from the full analysis, as the solid-state sensor demonstrated non-physiological signals (i.e., Pes swings exceeding Paw) and the Nutrivent catheter was unreliable due to balloon emptying despite recalibration attempts (Supplementary material 8). For controlled ventilation, one additional patient was excluded due to balloon (Cooper) catheter emptying (but included for assisted ventilation after adequate recalibration). For assisted ventilation, three additional patients were excluded (but included in controlled ventilation analysis) due to: technical issues (n = 1), very low breathing efforts (n = 1), many artifacts hampering breath detection (n = 1). This resulted in a total analysis set of 2200 breaths from 14 patients during controlled ventilation and 889 breaths from 12 patients during assisted ventilation; all Pes_bal_ tracings were adequately calibrated with a Baydur maneuver between 0.9–1.1. No adverse events were reported.

**Table 1:** Main characteristics of the study population

Characteristic	Total *(N* = *16)* (median (IQR) or number)
Age (years)	65 (58–69)
Males	15
BMI (kg/m^2^)	26.7 (25.0–29.7)
IBW (kg)	76.9 (68.1–79.2)
**Medical history**	
Asthma	1
Non-obstructive emphysema	1
Coronary artery disease	6
Renal Failure	2
**Type of surgery performed**	
CABG	5
CABG + AVR	2
ROSS procedure	2
MVR-MIC	3
AVR	4

	Controlled ventilation (median (IQR))	Assisted ventilation (median (IQR))
**Respiratory parameters**		
PEEP set (cmH_2_O)	7 (7–8)	7 (6–8)
PEEP total (cmH_2_O)	8.5 (7.7–9.1)	N/A
PC/PS above PEEP (cmH_2_O)	11 (10–13.3)	7 (5–8)
Pplat (cmH_2_O)	17 (16.2–19.7)	N/A
Driving Pressure (cmH_2_O)	9.5 (7.7–10.7)	N/A
Pocc (cmH_2_O)	N/A	12.7 (8.4–17.3)
RR (/min)	16 (16–18)	13 (9–15)
Tidal Volume (mL)	484 (448–568)	689 (459–856)
Minute Volume (L/min)	7.9 (7.0–8.7)	6 (5.6–7.3)
FiO_2_ (%)	30 (30–41)	35 (30–41)
SpO_2_ (%)	98 (97–98)	98 (97–99)
EtCO_2_ (kPa)	5.4 (4.6–5.9)	6.8 (5.8–7.3)
**Hemodynamic parameters**		
Heart rate (/min)	75 (68–81)	82 (79–90)
BP systolic (mmHg)	103 (94–109)	138 (122–154)
BP diastolic (mmHg)	56 (53–59)	65 (61–68)
MAP (mmHg)	70 (66–74)	85 (80–95)

AVR aortic valve replacement, BMI body mass index, BP Blood pressure, CABG coronary artery bypass graft, EtCO_2_ End-tidal CO_2_, FiO_2_ fraction of inspired oxygen, IBW ideal body weight, IQR interquartile Range, MAP Mean Arterial Pressure, MVR-MIC mitral valve repair with minimal invasive surgery, PC pressure control, PEEP positive end-expiratory pressure, PS Pressure Support, Pplat plateau pressure, Pocc occlusion pressure, ROSS procedure aortic valve replacement with own pulmonary valve and pulmonary allograft, RR Respiratory Rate, SpO_2_ oxygen saturation

### Signal interference

Signal interference between the solid-state and Nutrivent catheter is explained in Supplementary material 9, likely the result of having two rather thick catheters (with large balloon of Nutrivent catheter) in place and rendering parts of the data unusable for further analysis. After using the Cooper catheter (from the 5th patient), such interference was not observed.

### Comparisons

**Fig. 4:**
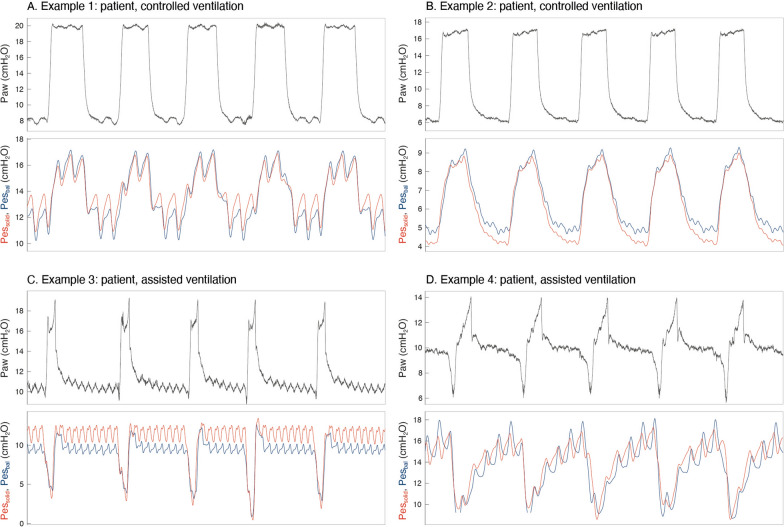
Examples of tracings obtained in four different patients during controlled ventilation (**A**, **B**) and assisted ventilation (**C**, **D**)

**Fig. 5:**
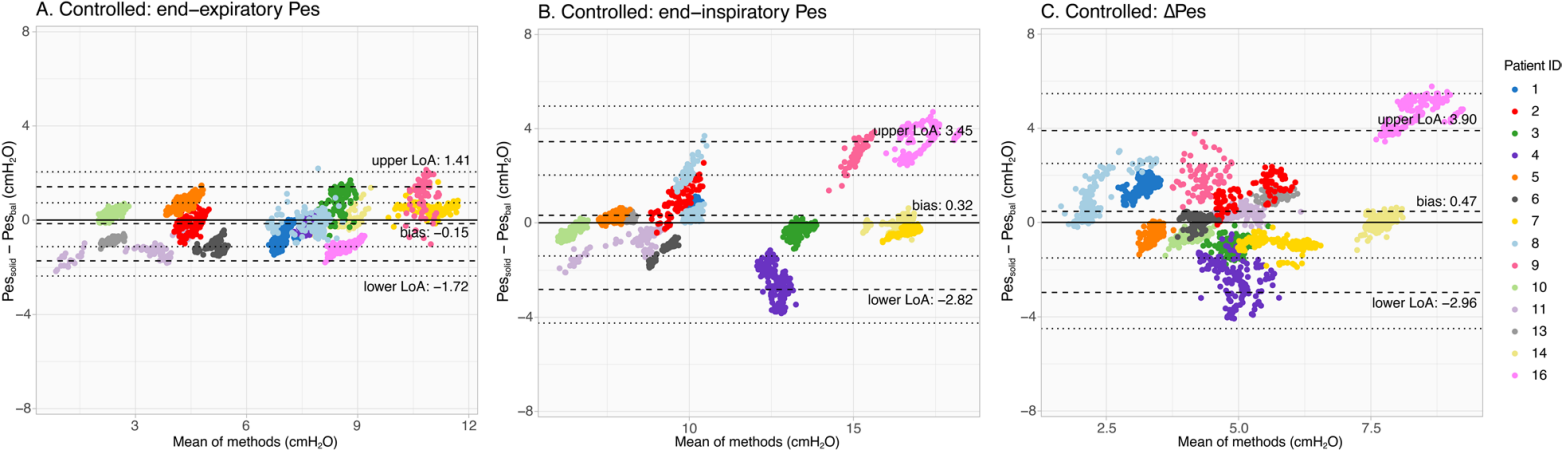
Bland–Altman results for patients on controlled ventilation for the end-expiratory (**A**), end-inspiratory (**B**) and delta (**C**) Pes values. All individual breaths are presented and each color represents a different patient. Patient ID is provided for comparison with Fig. 6. Dashed lines represent the bias and upper and lower limits of agreement (LoA); dotted lines represent the 95% confidence interval of the LoA obtained via bootstrapping

Figure 4 shows Pes_bal_ and Pes_solid_ tracings during controlled and assisted ventilation. During controlled ventilation, Bland–Altman analyses (Fig. 5A–C, Supplementary material 1: Table S2-A) revealed a low bias (i.e., Pes_solid_ – Pes_bal_)) [upper LoA; lower LoA] of − 0.15 [1.41; − 1.72], 0.32 [3.45; − 2.82] and 0.47 [3.90; − 2.96] cmH_2_O for end-expiratory, end-inspiratory and ΔPes values, respectively. Patient 16 demonstrated inspiratory pressure amplifications in Pes_solid_ that we could not attribute to cardiac artifacts solely (Supplementary material 10). Removing this patient improved comparisons (Supplementary material 1: Table S2-B): bias [upper LoA; lower LoA] of − 0.08 [1.45; − 1.60], 0.07 [2.70; − 2.57] and 0.15 [2.71; − 2.42] cmH_2_O for end-expiratory, end-inspiratory and ΔPes values, respectively.

Pressures obtained during static conditions are presented in Table 2; LoA were smaller (all ≤ 2 cmH_2_O) as compared to breath-by-breath analysis.

**Table 2:** Static comparisons for Pes_solid_ – Pes_bal_ (controlled ventilation)^1^

	Expiratory hold	Inspiratory hold	ΔPes
Mean difference (cmH_2_O)	−0.21	−0.36	−0.15
SD of difference (cmH_2_O)	0.93	0.79	0.78
Upper LoA (cmH_2_O)	1.61	1.19	1.37
Lower LoA (cmH_2_O)	−2.02	−1.90	−1.67

^1^Patient 16 was removed from this analysis due to unphysiological inspiratory and delta Pes values

**Fig. 6:**
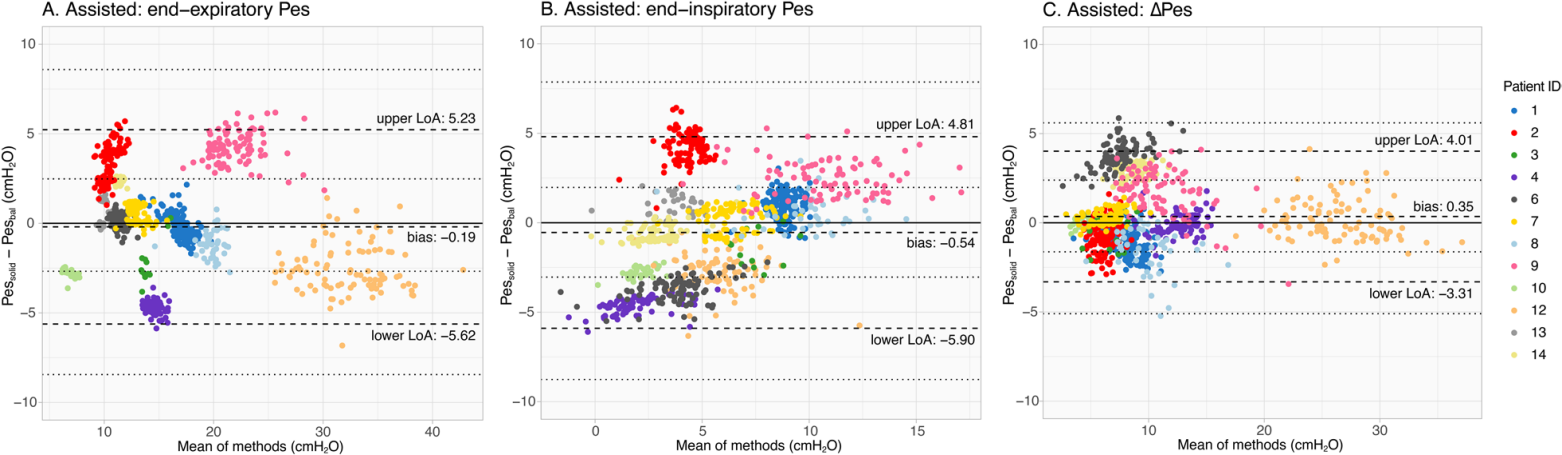
Bland–Altman results for patients on assisted ventilation. All individual breaths are presented and each color represents a different patient; subject’s color coding is similar for Fig. 5. Dashed lines represent the bias and upper and lower limits of agreement (LoA); dotted lines represent the 95% confidence interval of the LoA obtained via bootstrapping

During assisted ventilation, bias remained low, but LoAs were wider, yet smaller than in healthy volunteers (Fig. 6, Supplementary material 1: Table S2-C): − 0.19 [5.23; − 5.62], − 0.54 [4.81; − 5.90] and 0.35 [4.01; − 3.31] cmH_2_O for end-expiratory, end-inspiratory and ΔPes values, respectively.

A total of 24 Baydur maneuvers from the solid-state catheter were analyzed to determine sensor stability (for n = 12 patients in controlled ventilation (2 missing as acquisition started after balloon calibration) and for n = 12 patients in assisted mode). The mean ∆Pes_solid_/∆Paw ratio was 1.05 ± 0.18 [min–max: 0.72–1.48].

## Discussion

We tested a new Pes catheter with solid-state sensor on the bench, in healthy volunteers and in ventilated patients. Findings can be summarized as: 1) the solid-state sensor demonstrated excellent agreement with a reference pressure during a 5-day bench test, without signal drift; 2) good to excellent agreements with Pes_bal_ during tidal breathing was found in healthy volunteers when Pes_bal_ was adequately calibrated; 3) these agreements improved in ventilated patients during tidal breathing (controlled and assisted ventilation) and further in static conditions (breath holds); 4) the sensor remained stable throughout study recordings; 5) the solid-state Pes catheter often measured higher ΔPes values, especially in healthy volunteers during high efforts.

### Validity of reference pressure

For our in-human comparisons, balloon Pes catheters served as “reference standard” as the true pleural pressure is not available in humans. A concern with this approach in our and previous studies[7–10] is that each balloon has an optimal filling volume, depending on its perimeter/length, and elasticity and length of connecting tubing. These characteristics, but also the balloon’s position in the esophagus and ex-vivo pressure sensor affect absolute pressure values and the balloon’s capacity to respond to Pes swings (i.e., frequency response)[11]. Furthermore, the balloon may empty over time and recommended filling volumes by manufacturers are often not optimal clinically; changes in intrathoracic pressure or chest wall compliance (e.g. change in PEEP, body position, pleural pressure inhomogeneities) require recalibration[1, 3, 11, 12]. Indeed, uncalibrated and calibrated balloon pressures (i.e., corrected for esophageal wall and balloon elasticity) could differ at end-expiration by 5.1 cmH_2_O (range: 0.8 to 35.1 cmH_2_O) despite obtaining a Baydur range of 0.8–1.2 [11]. This makes the use of balloon catheters as reference standard somewhat questionable. We aimed to target higher balloon catheter accuracy for primary comparisons (ΔPes_bal_/ΔPaw within 0.9–1.1 range) to improve comparisons, but this was sometimes challenging in healthy volunteers. A Baydur range of 0.8–1.2 is considered acceptable in literature[1] but also implies an accepted deviation of 20% from the true ∆Pes value, which may impact clinical decision-making when applying Pes-based ventilation strategies. Considering that the solid-state sensor had an excellent offline obtained ΔPes_solid_/ΔPaw (1.01 ± 0.14 in healthy volunteers, 1.05 ± 0.18 in patients) and bench results, this sensor was stable in providing a biased (delta) Pes from the balloon catheter. It can also be argued that the solid-state sensor better represented the actual pressures; this is especially relevant in the presence of large inspiratory efforts, where balloon catheter-tubing systems might be too slow to follow rapid pressure changes. Studies comparing different Pes sensor types (i.e., solid-state, balloons [12], liquid-filled catheters [13, 14]) are therefore challenging to interpret without the clinical availability of a true reference standard.

### Related works

Over 20 years ago, solid-state Pes catheters were compared with a balloon catheter in healthy volunteers[7, 9] demonstrating reliable relative/delta Pes values. However, uncontrollable offset shifts (10 cmH_2_O for transpulmonary pressure[7]) and a high bias (> 7 cmH_2_O[9]) were observed, making absolute values unreliable. Authors hypothesized that Van der Waal forces contributed to falsely high pressures (e.g., mucus sticking to sensor membrane, or contact with the esophageal wall)[7] and negative signal drifts[9]. More recent work in 2017[8] and 2021[10] comparing micro-transducers with balloon catheters report a smaller bias: end-expiratory Pes of − 3.6 cmH_2_O[8] (vs. 1.6 cmH_2_O in our study) and delta Pes of 3.8 cmH_2_O[10] (vs. 3.9 cmH_2_O in our study), respectively.

The smaller[7, 9] or comparable[8, 10] biases in our healthy volunteers can be explained as follows. First, our solid-state catheter includes a small balloon above the sensor serving as a stabilizer to avoid sensor sticking to the esophageal wall; this likely also kept mucus off the sensor membrane, avoiding signal drifts. Second, the sensor is both temperature and humidity calibrated. Yet, in some subjects/patients large differences with Pes_bal_ were found, which may be explained by the sensor’s fast frequency response: since pressures are measured directly inside the esophagus signal dampening is avoided, but artifacts can be easily amplified. Cardiac artifacts in Pes_solid_ were sometimes high and body position dependent (e.g., more marked in supine position, see Supplementary material 11, in line with[9]), and more negative Pes values were observed with larger inspiratory efforts (Fig. 2). This warrants careful identification and interpretation of artifacts, improved signal filtering at the bedside, and/or optimizing the sensor positioning. The smaller biases and LoA in ICU patients as compared to healthy volunteers could be explained by the stable ventilator settings and the low variability in breathing efforts during assisted mode. In addition, body position alterations were not part of the patient study.

Within-subject variability exists in pressures over the length of the esophagus [15–17], probably due to the gravitational effects of the lung on the pleural pressure and the anatomical position of the esophagus in the thoracic cavity (e.g., pressure of the heart on the esophagus in the lower third of the thorax). To minimize variability in measurement location between the solid-state sensor and balloon, we inserted the solid-state sensor at the depth corresponding to approximately halfway the balloon that was placed in the mid-esophageal range as per current practice. However, sensors (balloon vs. solid-state) may not have measured the pressure at exactly the same location within the esophagus, which cannot be confirmed at the bedside nor offline. For balloon catheters, the balloon surrounding the catheter has the function of ensuring that the holes in the catheter below the balloon do not occlude. Effectively, there is only one balloon hole that contributes to the pressure measured by the extracorporeal pressure sensor. Depending on external factors, balloon holes can become occluded, impacting the absolute and relative pressures measured. Eventually, the insertion depth needs to be standardized.

### *Strengths and limitations*

This is the first study validating a novel solid-state Pes catheter, combining bench work with measurement in healthy volunteers and ICU patients during different ventilation modes. The use of the solid-state Pes catheter was considered easy: it requires only one calibration prior to insertion and pressures are measured directly in the esophagus – hence, some secondary limitations of balloon catheters (e.g., need for precise filling volume, risks of balloon emptying over time) are not applicable. In contrast, with the sensor’s fast response time, there is a possibility of pressure amplifications related to e.g., cardiac artifacts as discussed above. Our study also has some limitations. First, we used two different balloon catheters in the patient study, which was initially designed with the Nutrivent catheter as comparator, but significant interference was observed. We also did not use the Vbest method via pressure–volume curves for balloon filling, as this method is rather cumbersome clinically, not applicable to non-ventilated healthy subjects and patients on assisted ventilation due to variety in respiratory effort (volumes), and there is no clinical evidence it results in better data/outcomes. Instead, for consistency across measurement conditions/populations, we used the manufacturer’s recommended filling volume as starting point and adjusted the filling volume according to the Baydur occlusion test, aiming for a tight range of 0.9–1.1. Second, several short sections were excluded from the analysis due to artifacts in both signals and/or unreliable Pes_bal_ tracings. Nevertheless, when selecting stable tracings where the balloon was properly calibrated, good agreement between Pes_bal_ and Pes_solid_ was found. Since our sample size was larger than necessary, excluding some tracings likely did not affect power of our analyses. Third, in healthy volunteers we measured positive and high end-expiratory Pes values, likely the result of inspiratory effort maneuvers and/or cardiac artifacts. In addition, two patients demonstrated unphysiologically high Pes_solid_ values (see Results) despite proper calibration, reposition attempts and verification of esophageal positioning. This warrants good instructions and training for standardized and reliable use during technology implementation. Last, we did not perform multiple-day testing of the solid-state catheter in the ICU, but extensive bench tests demonstrated only minimal signal drift over 5 days.

### Clinical relevance

Pes monitoring allows individualization of ventilator settings via a more thorough understanding of the mechanical properties of the respiratory system. Over the last years, use of the balloon catheters has improved with the availability of dedicated monitors or ventilator connections. Yet, measurements remain technically challenging and time-consuming, hampering widespread routine implementation [5]. Easy-to-use devices and protocols on the use, patient selection and interpretation of Pes tracings are needed [5]. The solid-state catheter requires only one calibration prior to insertion, contributing to its ease of use. This makes the technique interesting for future implementation, also in the light of the recent regulatory challenges such as the medical device regulation in the European Union, which has put extensive pressure on the production of medical devices, resulting in limited availability and even withdrawal of certain balloon catheters from the market. Future work should focus on the longer-term use of the technique, e.g., in multiple-day measurements within a Pes-guided ventilation strategy, and optimizing filtering of (cardiac) artifacts and (automated) signal quality checks.

In conclusion, this is the first study validating a novel solid-state Pes catheter in vitro, in healthy volunteers and in postoperative mechanically ventilated ICU-patients, with promising results. This could contribute to the implementation of Pes as advanced respiratory monitoring technique.

## Supplementary Information

Supplementary material 1

Supplementary material 2

Supplementary material 3

Supplementary material 4

Supplementary material 5

Supplementary material 6

Supplementary material 7

Supplementary material 8

Supplementary material 9

Supplementary material 10

Supplementary material 11

## Data Availability

No datasets were generated or analysed during the current study.

## Abbreviations

BMI: Body Mass Index
ICU: Intensive Care Unit
iEPC: Intelligent Esophageal Pressure Catheter
iEPMS: Intelligent Esophageal Pressure Monitoring System
LoA: Limit of Agreement
Paw: Airway pressure
Pes: Esophageal pressure
Pes_bal_: Esophageal pressure measured by balloon catheter
P_ref_: Reference pressure
P_solid_: Esophageal pressure measured by the solid-state pressure sensor
