# Supplementary material for: Solid-state esophageal pressure sensor for the estimation of pleural pressure: a bench and first-in-human validation study
Source: Crit Care. 2025 Jan 27;29:47. doi: 10.1186/s13054-025-05279-w (PMC11773869; doi:10.1186/s13054-025-05279-w)
Supplement: Supplementary file 1 — Supplementary material 1 [file 13054_2025_5279_MOESM1_ESM.docx]

*Additional file 1 to:*

**Solid-state esophageal pressure sensor for the estimation of pleural pressure: a bench and first-in-human validation study**

Julien P. van Oosten^1^, Nico Goedendorp^1^, Amne Mousa^2,3^, Rutger C. Flink^4^, Rik Schaart^4^,

Merel Flinsenberg^1^, Peter Somhorst^1^, Diederik A.M.P.J. Gommers^1^, L.M.A. Heunks^5^,

Annemijn H. Jonkman^1^

**Author affiliations:**

1. Intensive Care, Erasmus Medical Center, Rotterdam, The Netherlands
2. Intensive Care, Amsterdam UMC location Vrije Universiteit Amsterdam, The Netherlands
3. Amsterdam Cardiovascular Sciences, Amsterdam, the Netherlands
4. Pulmotech B.V., Leek, The Netherlands
5. Intensive Care, Radboud University Medical Center, Nijmegen, The Netherlands

ADDITIONAL METHODS

Calibration of the solid-state Pes catheter

Prior to insertion and when connected to the acquisition device, the solid-state sensor was calibrated: two layers of sterile gauze pad soaked in water were placed on the sensor to create a humid environment. After 2 minutes the zero button was pressed and it was verified that pressures were 0 cmH_2_O.

***Healthy volunteers***

Baydur test

Subjects were breathing through a flow-sensor connected to a mouthpiece while wearing a nose clip. One limb of the flow sensor (differential pressure) was used to acquire airway pressure. The distal part of the flow sensor should be occluded during the Baydur test. This can be done e.g., with a hand, or using a loading device with maximum resistance (fully occluded) as illustrated below. An example of a Baydur measurement is shown (ΔPes_bal_/ΔPaw = 1.08).

| 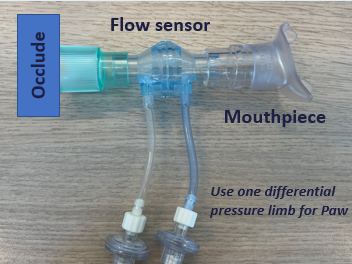 | 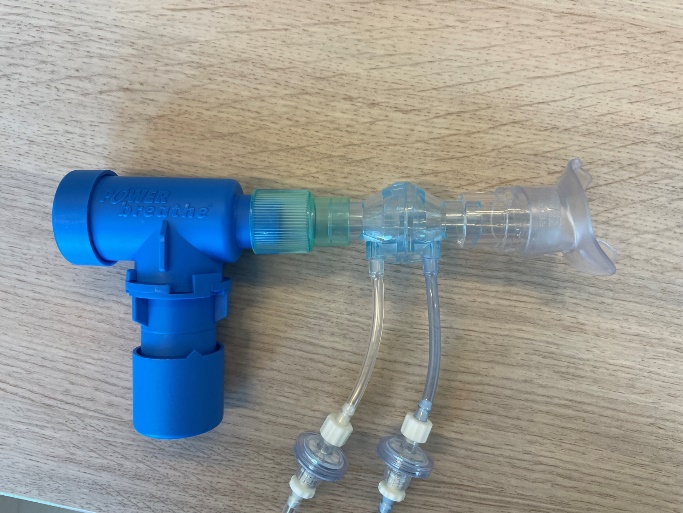  Occluded threshold loading device |
| --- | --- |
| 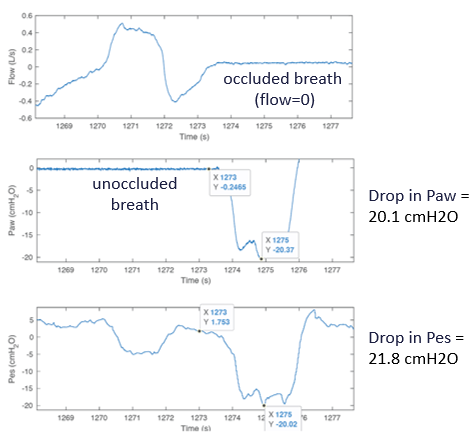 | |

**Additional figure 1.** The loading device to perform a Baydur test in healthy volunteers with an example of a Baydur measurement.

***Inspiratory loading protocol***

During sitting and semi-recumbent position, subjects were additionally exposed to three levels of inspiratory effort to obtain a variable within-subject range of effort and thus Pes values: medium loading (30% of maximum inspiratory pressure (PImax)), high loading (60% PImax) and maximum loading (90% of PImax). PImax was assessed prior to study recordings with maximum inspiratory effort maneuvers. Loading was applied by letting the volunteer breathe through a mouthpiece connected to a threshold loading device (Power Breathe, POWERbreathe Ltd, UK) while wearing a nose-clip. The duration of the loading task was decreased as load increased: 2 minutes quiet breathing – 45 seconds medium loading – 1 minute rest – 30 seconds high loading – 1 minute rest – 15 seconds maximum loading.

***Statistical analysis***

Statistical analysis was performed in R (version 4.4.1; R Foundation for Statistical Computing, Vienna, Austria) using the packages tidyverse, ggplot2, dplyr, pals, lme4, MuMIn, nlme and rmba.R.

Different analyses were performed comparing Pes values between methods.

- *Bench test:* bias (i.e., P_solid_ – P_ref_), standard deviation (SD) of the differences, and limits of agreement (i.e., bias ± 1.96 SD) were computed and visualized per measurement time point, separately for the minimum, maximum and delta pressures. As we were interested in the change in bias over time (5 days), we obtained the bias and LoA for each individual time point. In addition, considering the dependency between repeated measures of the catheters, we performed a linear mixed effects model to obtain the overall within-sensor variability (i.e., residual SD) and between-sensor variability (i.e., random effects SD) of the bias over the full 5 days. The model was implemented (lme4 package) as *model = lmer(differences ~ 1 + (1 | catheter), data = data_bench)*. Here, the dependent variable is the difference between P_solid_ and P_ref_, the fixed effect represents the overall bias, and we implemented a random intercept for each catheter.
- *Breath-by-breath analysis in healthy subjects and patients:* Bland-Altman analysis was performed to evaluate the bias of the solid-state catheter, separately for the end-expiratory, end-inspiratory and delta pressures. As several pairs of measurements were taken by each subject and classic Bland-Altman analysis assumes independent observations, we corrected for the repeated measures design using mixed models. We accounted for the different number of measurements between subjects (e.g. due to varying respiratory rates and included breaths with sufficient signal quality) by using bootstrap resampling with 1000 iterations to obtain the 95% confidence intervals of the bias, SD and LoAs. Both bootstrapping and mixed effects models were implemented via the rmba package in R, that is based on the work of Parker et al. [1] and Thai et al. [2].
- *Static measurements in patients (three end-expiratory and end-inspiratory holds during controlled ventilation):* Bland-Altman analysis was performed to compute the mean difference, SD of differences and 95% LoAs via linear mixed effects models to account for the repeated measures design, but without bootstrap resampling, as the number of data points was identical between patients. This was also computed via the rmba package.
- For the healthy volunteers where variability in inspiratory effort was introduced with loaded breathing, agreement between Pes values for the solid-state vs. balloon was evaluated with a linear mixed effects model (lme4 package) as: *fit = lmer(Pes_balloon ~ Pes_solid + (1 + Pes_solid | subject), data=data_healthy)*. This was performed three times for the end-expiratory, end-inspiratory and delta Pes values separately. For our random effects structure, the maximal random effects approach using (1 + Pes_solid | subject) was chosen to account for all sources of non-independence from repeated measures from the same subject. The conditional R^2^ was obtained using the MuMIn package. Correlations for each individual patient were also computed with simple linear regression.

ADDITIONAL RESULTS

**Table S1.** Bland-Altman results with bootstrapping confidence intervals (CI) of the bias, standard deviation (SD) and lower and upper limits of agreement (LoA) for healthy volunteers data.

| **A. Healthy volunteers – primary analysis (563 breaths)** | | | | |
| --- | --- | --- | --- | --- |
|  | bias | SD | upper LoA | lower LoA |
| End-expiratory Pes | 1.59 | 3.38 | 8.21 | -5.02 |
| 95% CI lower | 0.60 | 3.04 | 6.91 | -6.24 |
| 95% CI upper | 2.56 | 3.81 | 9.44 | -3.77 |
| End-inspiratory Pes | -2.32 | 3.36 | 4.27 | -8.92 |
| 95% CI lower | -3.40 | 2.96 | 2.90 | -10.34 |
| 95% CI upper | -1.22 | 3.79 | 5.65 | -7.56 |
| ΔPes | 3.91 | 3.64 | 11.04 | -3.23 |
| 95% CI lower | 2.91 | 3.30 | 9.80 | -4.50 |
| 95% CI upper | 4.94 | 4.05 | 12.30 | -1.99 |
| **B. Healthy volunteers – sensitivity analysis 1: Baydur balloon 0.8-1.2 (877 breaths)** | | | | |
|  | bias | SD | upper LoA | lower LoA |
| End-expiratory Pes | 1.44 | 3.20 | 7.71 | -4.83 |
| 95% CI lower | 0.51 | 2.89 | 6.57 | -5.95 |
| 95% CI upper | 2.33 | 3.55 | 8.85 | -3.82 |
| End-inspiratory Pes | -2.21 | 3.04 | 3.74 | -8.17 |
| 95% CI lower | -2.91 | 2.82 | 2.87 | -9.00 |
| 95% CI upper | -1.49 | 3.32 | 4.57 | -7.27 |
| ΔPes | 3.66 | 3.44 | 10.40 | -3.08 |
| 95% CI lower | 2.89 | 3.18 | 9.48 | -4.10 |
| 95% CI upper | 4.45 | 3.76 | 11.29 | -2.12 |
| **C. Healthy volunteers – sensitivity analysis 2: Baydur both catheters 0.9-1.1 (357 breaths)** | | | | |
|  | bias | SD | upper LoA | lower LoA |
| End-expiratory Pes | 0.70 | 3.19 | 6.95 | -5.55 |
| 95% CI lower | -0.52 | 2.69 | 5.29 | -7.27 |
| 95% CI upper | 2.03 | 3.83 | 8.68 | -3.87 |
| End-inspiratory Pes | -2.12 | 2.81 | 3.38 | -7.62 |
| 95% CI lower | -3.19 | 2.32 | 1.89 | -9.25 |
| 95% CI upper | -0.98 | 3.37 | 4.95 | -6.17 |
| ΔPes | 2.86 | 2.89 | 8.52 | -2.80 |
| 95% CI lower | 2.16 | 2.64 | 7.64 | -3.70 |
| 95% CI upper | 3.54 | 3.18 | 9.46 | -1.91 |

**Table S2.** Bland-Altman results with bootstrapping confidence intervals (CI) of the bias, standard deviation (SD) and lower and upper limits of agreement (LoA) for breath-by-breath data of patients.

| **A. Controlled ventilation – primary analysis (2200 breaths)** | | | | |
| --- | --- | --- | --- | --- |
|  | bias | SD | upper LoA | lower LoA |
| End-expiratory Pes | -0.15 | 0.80 | 1.41 | -1.72 |
| 95% CI lower | -0.56 | 0.55 | 0.73 | -2.36 |
| 95% CI upper | 0.24 | 1.06 | 2.05 | -1.12 |
| End-inspiratory Pes | 0.32 | 1.60 | 3.45 | -2.82 |
| 95% CI lower | -0.45 | 1.02 | 2.03 | -4.25 |
| 95% CI upper | 1.15 | 2.25 | 4.96 | -1.39 |
| ΔPes | 0.47 | 1.75 | 3.90 | -2.96 |
| 95% CI lower | -0.42 | 1.14 | 2.29 | -4.40 |
| 95% CI upper | 1.36 | 2.37 | 5.32 | -1.38 |
| **B. Controlled ventilation – without patient 16 (2008 breaths)** | | | | |
|  | bias | SD | upper LoA | lower LoA |
| End-expiratory Pes | -0.08 | 0.78 | 1.45 | -1.60 |
| 95% CI lower | -0.44 | 0.53 | 0.84 | -2.27 |
| 95% CI upper | 0.31 | 1.06 | 2.12 | -0.97 |
| End-inspiratory Pes | 0.07 | 1.34 | 2.70 | -2.57 |
| 95% CI lower | -0.73 | 0.90 | 1.53 | -3.81 |
| 95% CI upper | 0.78 | 1.86 | 3.92 | -1.37 |
| ΔPes | 0.15 | 1.31 | 2.71 | -2.42 |
| 95% CI lower | -0.59 | 0.85 | 1.54 | -3.63 |
| 95% CI upper | 0.82 | 1.78 | 3.87 | -1.29 |
| **C. Assisted ventilation (889 breaths)** | | | | |
|  | bias | SD | upper LoA | lower LoA |
| End-expiratory Pes | -0.19 | 2.77 | 5.23 | -5.62 |
| 95% CI lower | -1.92 | 1.67 | 2.48 | -8.44 |
| 95% CI upper | 1.64 | 4.08 | 8.58 | -2.68 |
| End-inspiratory Pes | -0.54 | 2.73 | 4.81 | -5.90 |
| 95% CI lower | -2.30 | 1.58 | 1.97 | -8.76 |
| 95% CI upper | 1.19 | 3.94 | 7.86 | -3.04 |
| ΔPes | 0.35 | 1.87 | 4.01 | -3.31 |
| 95% CI lower | -0.79 | 1.25 | 2.39 | -5.10 |
| 95% CI upper | 1.42 | 2.55 | 5.60 | -1.63 |

*References*

1. Parker RA, Weir CJ, Rubio N, Rabinovich R, Pinnock H, Hanley J, McCloughan L, Drost EM, Mantoani LC, MacNee W, McKinstry B. Application of Mixed Effects Limits of Agreement in the Presence of Multiple Sources of Variability: Exemplar from the Comparison of Several Devices to Measure Respiratory Rate in COPD Patients. PLoS One. 2016 Dec 14;11(12):e0168321. doi: 10.1371/journal.pone.0168321.
2. Thai HT, Mentré F, Holford NH, Veyrat-Follet C, Comets E. A comparison of bootstrap approaches for estimating uncertainty of parameters in linear mixed-effects models. Pharm Stat. 2013 May-Jun;12(3):129-40. doi: 10.1002/pst.1561.
