# Supplementary material for: Solid-state esophageal pressure sensor for the estimation of pleural pressure: a bench and first-in-human validation study
Source: Crit Care. 2025 Jan 27;29:47. doi: 10.1186/s13054-025-05279-w (PMC11773869; doi:10.1186/s13054-025-05279-w)
Supplement: Supplementary file 2 — Supplementary material 2 [file 13054_2025_5279_MOESM2_ESM.docx]

**Additional file 2**


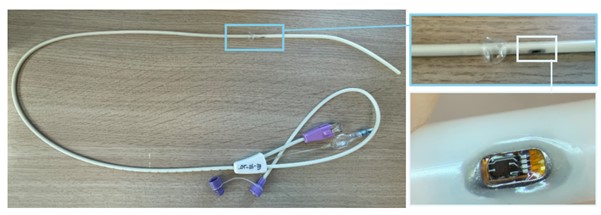


**Additional figure 2.** Intelligent esophageal pressure catheter (iEPC). The tiny solid-state sensor is embedded in the catheter, with a small balloon just above this sensor (top right photo). This balloon does not record any pressures but serves as a placeholder to make sure the sensor does not attach/stick to the esophageal wall.
