# Supplementary figures and images for: Solid-state esophageal pressure sensor for the estimation of pleural pressure: a bench and first-in-human validation study
Source: Crit Care. 2025 Jan 27;29:47. doi: 10.1186/s13054-025-05279-w (PMC11773869; doi:10.1186/s13054-025-05279-w)

**Additional file 3**

**
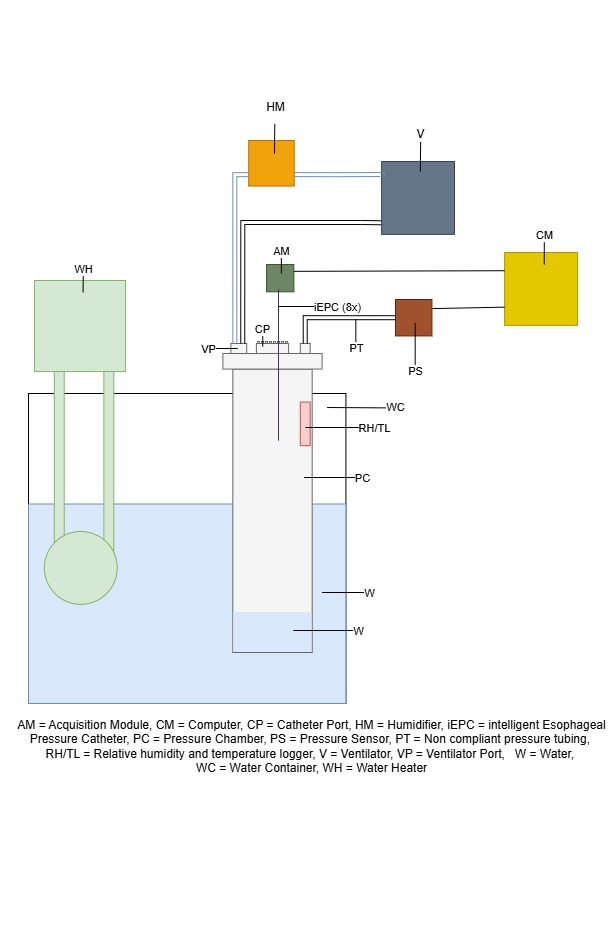
**

**Additional figure 3.** Bench setup for 5-day testing.

Supplement: Supplementary file 3 — Supplementary material 3 [file 13054_2025_5279_MOESM3_ESM.docx]
