# Supplementary material for: Solid-state esophageal pressure sensor for the estimation of pleural pressure: a bench and first-in-human validation study
Source: Crit Care. 2025 Jan 27;29:47. doi: 10.1186/s13054-025-05279-w (PMC11773869; doi:10.1186/s13054-025-05279-w)
Supplement: Supplementary file 6 — Supplementary material 6 [file 13054_2025_5279_MOESM6_ESM.docx]

**Additional file 6**

**
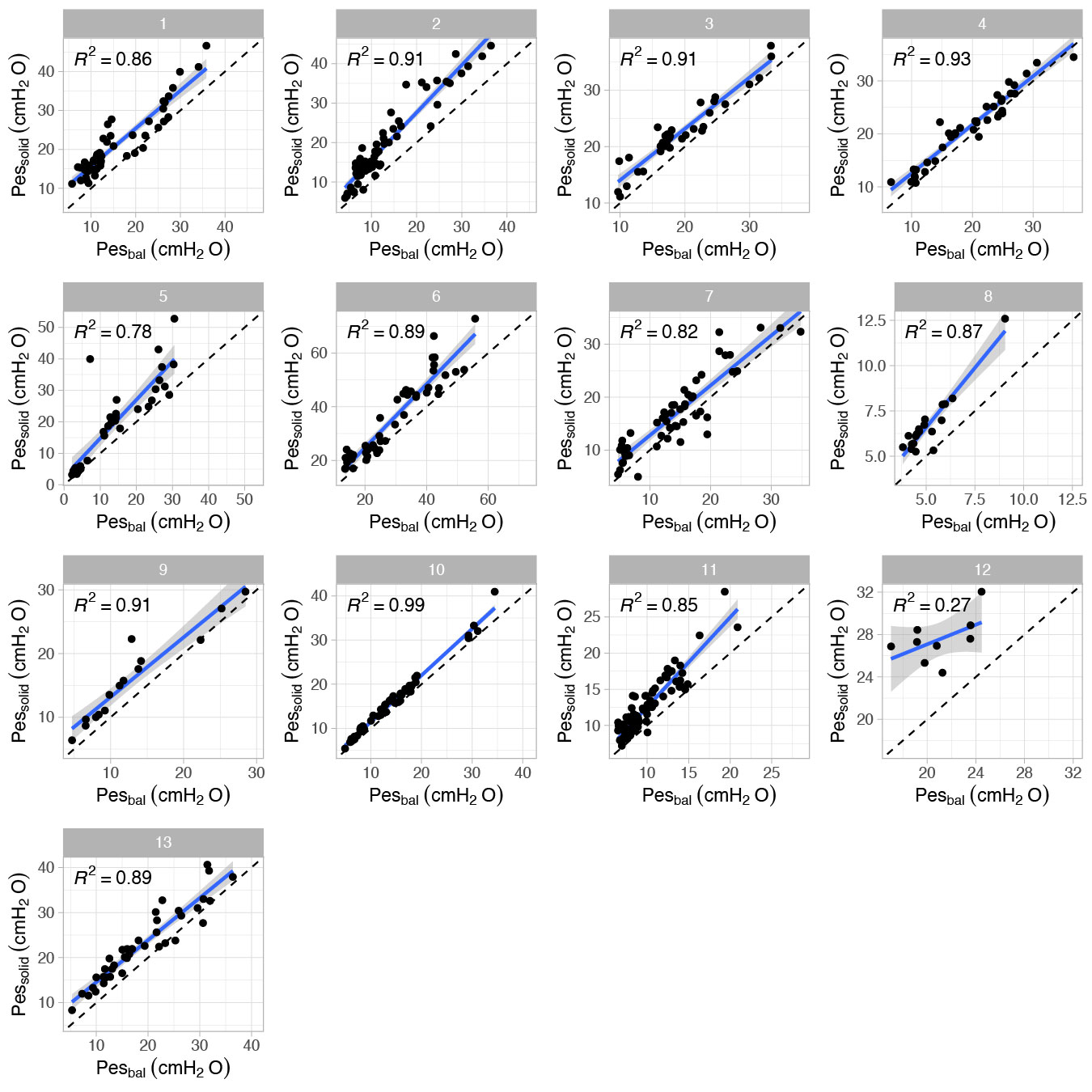
**

**Additional figure 6.** Healthy volunteers: regression analysis for ∆Pes values separated per subject.
