# Supplementary material for: Solid-state esophageal pressure sensor for the estimation of pleural pressure: a bench and first-in-human validation study
Source: Crit Care. 2025 Jan 27;29:47. doi: 10.1186/s13054-025-05279-w (PMC11773869; doi:10.1186/s13054-025-05279-w)
Supplement: Supplementary file 7 — Supplementary material 7 [file 13054_2025_5279_MOESM7_ESM.docx]

**Additional file 7**

**
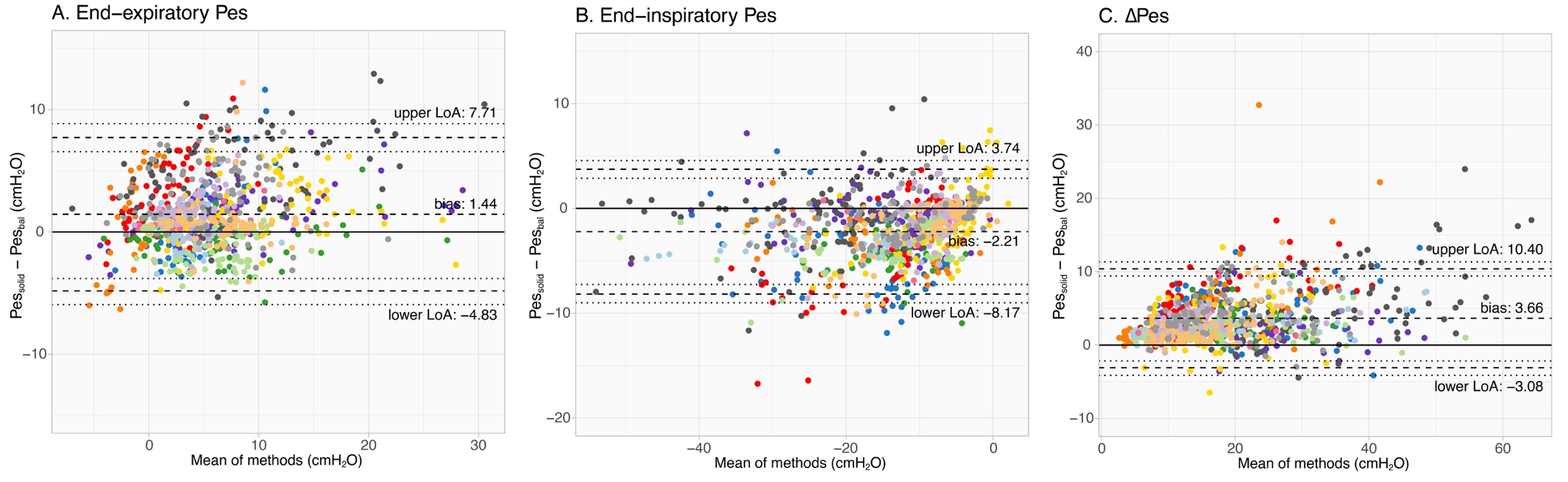
**

**Additional figure 7.** Healthy volunteers: Bland-Altman results for Baydur balloon within range 0.8-1.2. Each color represent a different subject. Dashed lines represent the bias and upper and lower limits of agreement (LoA); dotted lines represent the 95% confidence interval of the LoA obtained via bootstrapping.
