# Supplementary material for: Solid-state esophageal pressure sensor for the estimation of pleural pressure: a bench and first-in-human validation study
Source: Crit Care. 2025 Jan 27;29:47. doi: 10.1186/s13054-025-05279-w (PMC11773869; doi:10.1186/s13054-025-05279-w)
Supplement: Supplementary file 8 — Supplementary material 8 [file 13054_2025_5279_MOESM8_ESM.docx]

**Additional file 8**

**
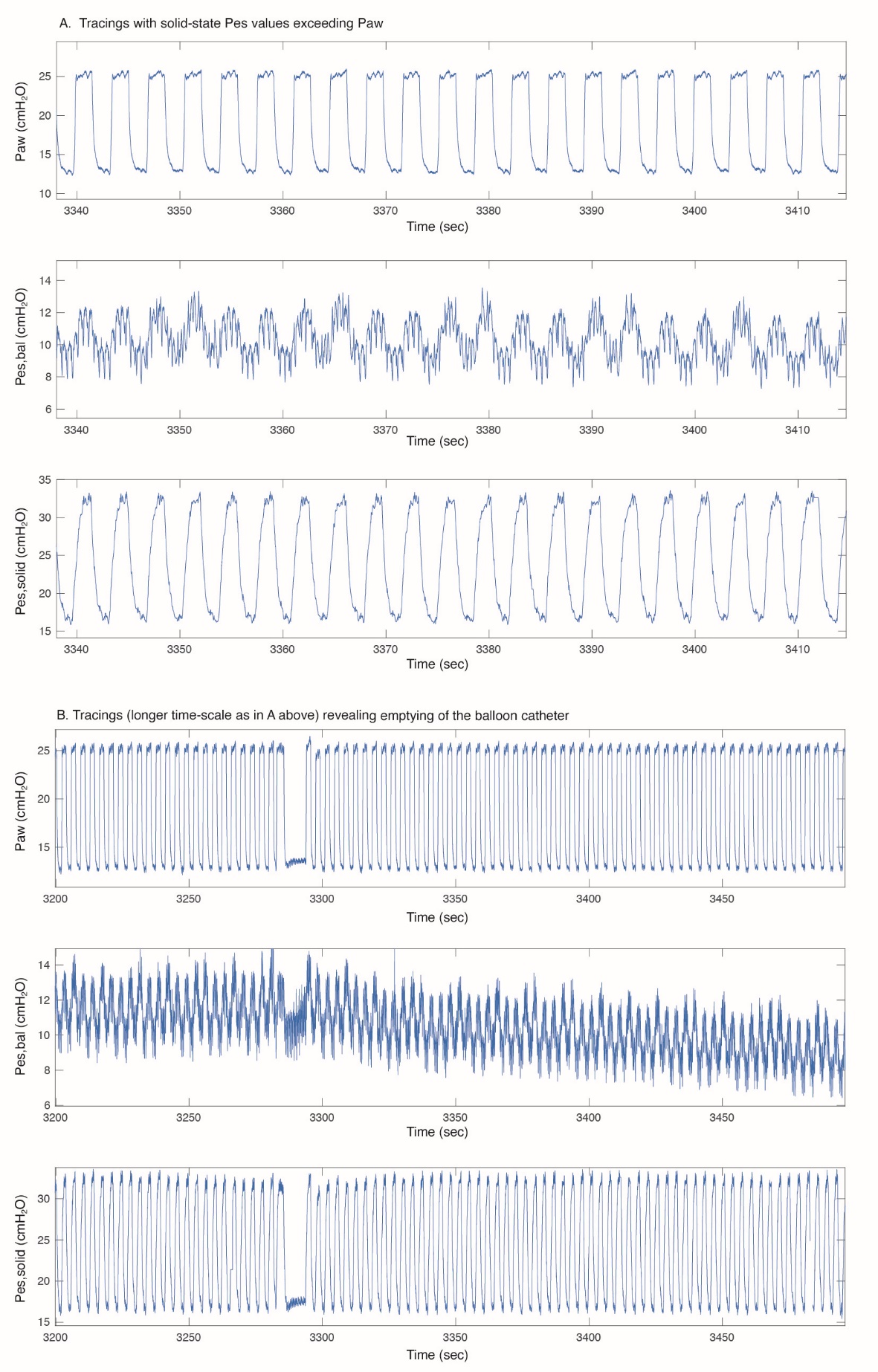
**

**Additional figure 8.** A. Signals from excluded patient as the solid-state sensor demonstrated non-physiological signals (i.e., Pes swings exceeding Paw). B. At the same time, the balloon catheter was unreliable due to emptying despite several recalibration attempts.
