# Supplementary material for: Solid-state esophageal pressure sensor for the estimation of pleural pressure: a bench and first-in-human validation study
Source: Crit Care. 2025 Jan 27;29:47. doi: 10.1186/s13054-025-05279-w (PMC11773869; doi:10.1186/s13054-025-05279-w)
Supplement: Supplementary file 9 — Supplementary material 9 [file 13054_2025_5279_MOESM9_ESM.docx]

**Additional file 9**

**
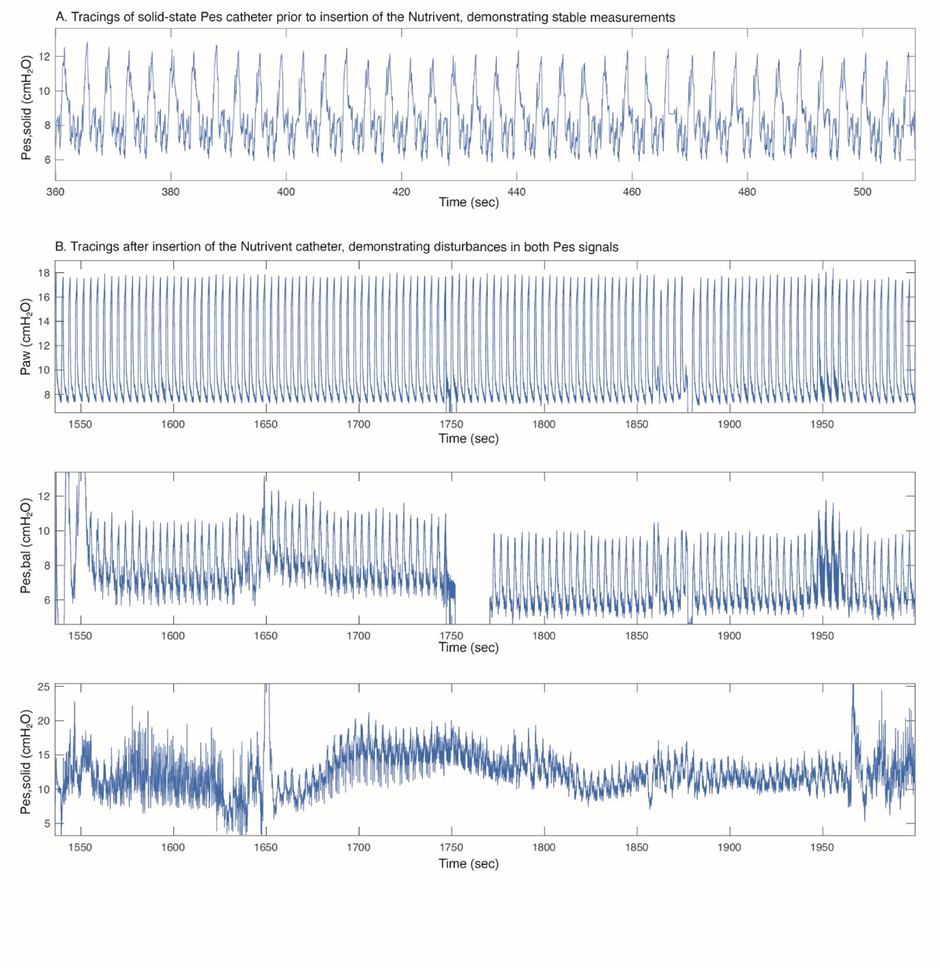
**

**Additional figure 9.** Signal interference between the solid-state and Nutrivent catheter. While Pes_solid_ demonstrated adequate signals prior to insertion of the balloon catheter (A), tracings became unstable afterwards (B). After several repositioning/refilling attempts of both catheters some stable parts were used for comparisons (not shown in this figure), despite artefacts in Pes_solid_. At 1750 seconds the balloon catheter briefly disconnected from the acquisition system (gap in recording).
