# Supplementary material for: Solid-state esophageal pressure sensor for the estimation of pleural pressure: a bench and first-in-human validation study
Source: Crit Care. 2025 Jan 27;29:47. doi: 10.1186/s13054-025-05279-w (PMC11773869; doi:10.1186/s13054-025-05279-w)
Supplement: Supplementary file 11 — Supplementary material 11 [file 13054_2025_5279_MOESM11_ESM.docx]

**Additional file 11.**

**
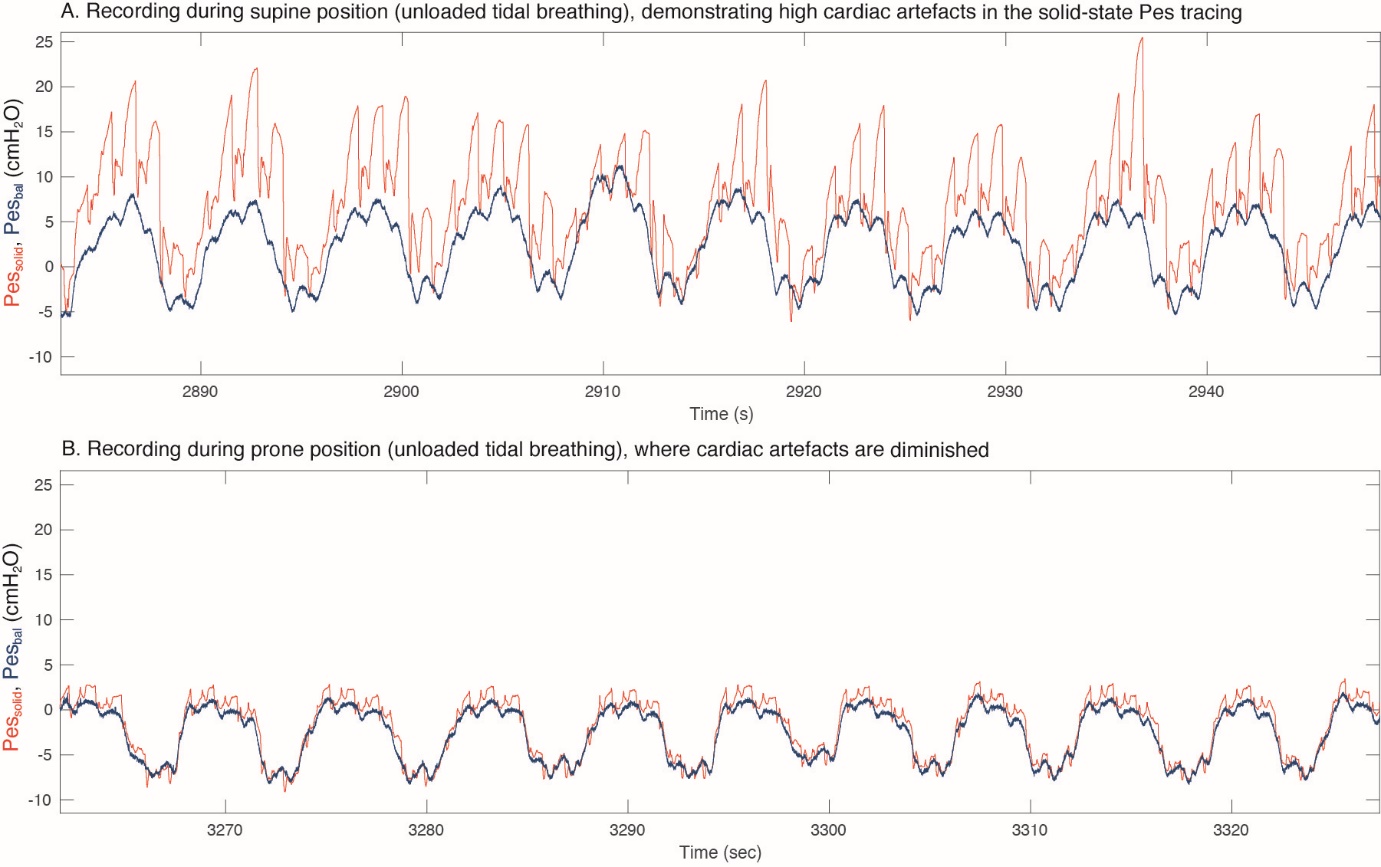
**

**Additional figure 11.** AB) Example of position-dependent artefacts in solid-state catheter
